# VOLUNTEERING AND RISK OF HEART ATTACK IN LATER LIFE: THE MODERATING ROLE OF PURPOSE IN LIFE?

**DOI:** 10.1093/geroni/igac059.2167

**Published:** 2022-12-20

**Authors:** Mallory Bell, Kenneth Ferraro

**Affiliations:** Purdue University, Center on Aging and the Life Course, Lafayette, Indiana, United States; Purdue University, West Lafayette, Indiana, United States

## Abstract

**Background and Objectives:**

Although research on the health benefits of volunteering has proliferated in recent decades, most studies are cross-sectional, and none prospectively examine the relationship between volunteering engagement, purpose in life, and heart attack.

**Research design and methods:**

This study uses seven waves of data (2006-2018) from the Health and Retirement study--a nationally representative survey of adults over age 50 (N=5,093). Event history analysis using Cox proportional hazards were used to examine if volunteering engagement in later life reduced the risk of first heart attack and if the effects of volunteering vary by level of purpose in life.

**Results:**

Results reveal that volunteering, and doing so at a low time commitment, reduces the risk of first heart attack in later life. Additionally, the effect of volunteering varies by sense of purpose in life, such that volunteers with a strong sense of purpose in life had the lowest risk of heart attack. Implications: The findings of this study will inform public health and policy interventions dedicated to extending and improving later life health.

